# Prevalence and trend of central nervous system–active medication polypharmacy among US commercially insured adults with vs without early-onset dementia: a multi-year cross-sectional study

**DOI:** 10.1186/s13195-024-01405-y

**Published:** 2024-02-08

**Authors:** Yu-Jung Jenny Wei, Nistha Shrestha, ChienWei Chiang, Steven T. DeKosky

**Affiliations:** 1 Division of Outcomes and Translational Sciences, College of Pharmacy, The Ohio State University, 500 West 12Th Avenue, Columbus, OH 43210-1291 USA; 2 Department of Biomedical Informatics, College of Medicine and Wexner Medical Center, The Ohio State University, Ohio, 43210 USA; 3 Department of Neurology and McKnight Brain Institute, University of Florida, Gainesville, FL 32610 USA

**Keywords:** Central nervous system-active medications, Early-onset dementia, Polypharmacy

## Abstract

**Background:**

Limited data exist on the prevalence and trend of central nervous system (CNS)-active medication polypharmacy among adults with early-onset dementia (EOD) and whether these estimates differ for adults without EOD but with chronic pain, depression, or epilepsy, conditions managed by CNS-active medications.

**Methods:**

A multi-year, cross-sectional study using 2012–2021 MarketScan Commercial Claims data was conducted among adults aged 30 to 64 years with EOD and those without EOD but having a diagnosis of chronic pain, depression, or epilepsy as comparison groups. For each disease cohort, the primary outcome was CNS-active medication polypharmacy defined as concurrent use of ≥ 3 CNS-active medications on the US Beers Criteria list that overlapped for > 30 consecutive days during 12 months following a randomly selected medical encounter with the disease diagnosis. A separate multivariate modified Poisson regression model was used to estimate time trends in CNS polypharmacy in each disease cohort. Differences in trend estimates between EOD and non-EOD disease cohorts were examined by an interaction between EOD status and yearly time.

**Results:**

From 2013 to 2020, the annual crude prevalence of CNS polypharmacy was higher among adults with EOD (21.2%–25.0%) than adults with chronic pain (5.1%–5.9%), depression (14.8%–21.7%), or epilepsy (20.0%–22.3%). The adjusted annual prevalence of CNS polypharmacy among patients with EOD did not significantly change between 2013 and 2020 (adjusted prevalence rate ratio [aPRR], 0.94; 95% CI, 0.88–1.01), whereas a significant decreasing trend was observed among non-EOD cohorts with chronic pain (aPRR, 0.66; 95% CI, 0.63–0.69), depression (aPRR, 0.81; 95% CI, 0.77–0.85), and epilepsy (aPRR, 0.86; 95% CI, 0.83–0.89). The interaction analysis indicated that patients with epilepsy and depression (vs with EOD) had a decreasing probability of CNS-active medication polypharmacy over time (aPRR, 0.98 [95% CI, 0.98–0.99]; *P* < .001 for interaction for both conditions).

**Conclusions:**

The prevalence of CNS polypharmacy among US commercially insured adults with EOD (vs without) was higher and remained unchanged from 2013 to 2021. Medication reviews of adults with EOD and CNS polypharmacy are needed to ensure that benefits outweigh risks associated with combined use of these treatments.

**Supplementary Information:**

The online version contains supplementary material available at 10.1186/s13195-024-01405-y.

## Introduction

Alzheimer’s disease and related dementia (ADRD), the most common neurodegenerative disorder, is expected to double in prevalence from 1.6% (5 million) in 2014 to 3.3% (14 million) by 2060 owing to the aging population of the United States (US) [1]. While mostly prevalent among individuals aged 65 years or older (i.e., late-onset dementia [LOD]), ADRD can develop before the age of 65 (i.e., early-onset dementia [EOD]). Early-onset dementia accounts for approximately 200,000 (4%) of more than 5 million Americans with dementia, with an estimated prevalence of 119 per 100,000 person-years in the US [2].

Central nervous system (CNS)-active medications are used to manage chronic neurological conditions (e.g., epilepsy), mental health conditions (e.g., depression and anxiety), and pain and sleep disorders, which are prevalent in patients with dementia [3–5]. Compared with patients with LOD, those with EOD were more likely to develop seizures [6] and neuropsychiatric symptoms, such as depression (41% vs 26%), and anxiety (28% vs 14%) [7]. Also, patients with EOD have a high prevalence of substance abuse disorder (e.g., alcohol dependence; 14.6%) and traumatic brain injury (6.4%) [8], strong predictors of persistent opioid use [9, 10]. Those conditions may predispose patients with EOD to the risk of using multiple CNS-active medications, or CNS medication polypharmacy. Concurrently using CNS-active medications, particularly long-term (e.g., > 30 days), has been associated with increased risks of falls [11], fall-related injury [12], and cognitive decline [13]. This evidence promoted the Beers Criteria from the American Geriatrics Society (AGS) to advise against concurrent use of three or more psychotropics and opioids for older adults, including those with dementia [14, 15]. While persons with dementia, including both LOD and EOD, are susceptible to serious drug adverse events associated with CNS-active medication polypharmacy, it is unclear the extent to which such potentially inappropriate prescribing practice is present among adults with EOD.

The current literature regarding investigations of CNS-active medication polypharmacy is primarily focused on patients with LOD who reside in nursing homes [16] or communities [17], older adults with depression [18], and adults [19] or older adults in general [20, 21]. Although valuable, those data have limited generalizability to patients with EOD. Using a US population-based health claims database from 2012 to 2021, this study has two aims: (1) to determine the prevalence and trend of CNS-active medication polypharmacy among US adults with EOD; and (2) to compare the prevalence and trend estimates among adults with EOD vs without EOD who had a diagnosis of chronic pain, depression, and epilepsy, conditions commonly managed through CNS-active medications.

## Methods

### Study design and source

This multi-year, cross-sectional study analyzed data from the 2012 through 2021 MarketScan Commercial Claims Database, which includes patients and dependents who receive employer-sponsored health insurance coverage from medium to large firms in the US [22]. The data contain individual billing records for inpatient and outpatient encounters and pharmacy-filled prescriptions, demographic characteristics, and enrollment status. The MarketScan claims databases have 56 million unique adult patients between the studied years, and MarketScan data have been widely used to study population-based trends in the use of various medications, including opioids [23], Z-drugs (e.g., zolpidem) [24], and antipsychotics [25]. The Ohio State University institutional review and privacy boards exempted this study from review because the data are de-identified.

### Study sample

To assemble the cohort, we identified patients aged 30–64 years who had at least one encounter from inpatient or outpatient claims with an *International Classification of Diseases, Ninth or Tenth Revision Clinical Modification* (*ICD-9-CM/ICD-10-CM)* code for ADRD (Table A. 1). These codes were used in a prior study identifying patients with LOD [20] and commonly used in various algorithms identifying patients with ADRD [26]. For each patient, a single medical encounter with ADRD (i.e., index date) that had continuous enrollment in healthcare insurance for 12 months before and after the encounter was randomly selected to avoid oversampling individuals and to obtain a representative sample of patients with EOD. We used the 12 months before the selected ADRD encounter to measure baseline variables (detailed in Sect. 2.5) and the 12-month period after the encounter to measure the key dependent variable—CNS-active medication polypharmacy (detailed in Sect. 2.4).

**Table 1:** Demographic and clinical characteristics of commercially insured adults with early-onset dementia overall and by receipt of central nervous system polypharmacy

**Characteristic**	Overall sample No. (%)	With CNS polypharmacy No. (%)	Without CNS polypharmacy No. (%)	*P* value
**Overall**	37,563 (100)	8,960 (100)	28,603 (100)	
**Age, y**				.019
Mean (SD)	55.9 (7.0)	56.0 (6.9)	55.9 (7.1)	
≤ 44	3532 (9.4)	777 (8.7)	2755 (9.6)	
45–49	3463 (9.2)	839 (9.4)	2624 (9.2)	
50–54	5764 (15.3)	1373 (15.3)	4391 (15.4)	
55–59	9468 (15.6)	2346 (26.2)	7122 (24.9)	
60–64	15336 (40.8)	3625 (40.5)	11,711 (40.9)	
**Sex**				< .001
Male	16,279 (43.3)	3225 (36.0)	13,054 (45.6)	
Female	21,284 (56.7)	5735 (64.0)	15,549 (54.4)	
**Locale**				.078
Metropolitan	27,138 (72.3)	6408 (71.5)	20,730 (72.5)	
Rural	10,425 (27.8)	2552 (28.5)	7873 (27.5)	
**US Region**				< .001
Northeast	7618 (20.3)	1473 (16.4)	6145 (21.5)	
Midwest	8025 (21.4)	1965 (21.9)	6060 (21.2)	
South	17,316 (46.1)	4496 (50.2)	12,820 (44.8)	
West	4604 (12.3)	1026(11.5)	3578 (12.5)	
**Clinical condition**				
Depression	18,019 (48.0)	7048 (78.7)	10,971 (38.4)	< .001
Psychotic disorder	2642 (7.0)	1235 (13.8)	1407 (4.9)	< .001
Behavioral symptoms	7361 (19.6)	2679 (29.9)	4682 (16.4)	< .001
Chronic pain	16,346 (43.5)	5326 (59.4)	11,020 (38.5)	< .001
Epilepsy	4327 (11.5)	1742 (19.4)	2585 (9.0)	< .001
Injury	3916 (10.4)	1500 (16.7)	2416 (8.5)	< .001
Substance use disorder	4459 (11.9)	1897 (21.2)	2562 (9.0)	< .001
CCI (Mean ± SD)	2.2 ± 2.0	2.7 ± 2.1	2.0 ± 1.9	< .001
**Subtype of indexed ADRD diagnosis**				< .001
Dementia with Lewy bodies	495 (1.3)	145 (1.6)	350 (1.2)	
Frontotemporal dementia	1160 (3.1)	246 (2.8)	914 (3.2)	
Vascular dementia	3132 (8.3)	821 (9.2)	2303 (8.1)	
AD/senile dementia	5333 (14.2)	965 (10.7)	4368 (15.3)	
Dementia unspecified	27451 (73.1)	6783 (75.7)	20668 (72.3)	
**Year of indexed ADRD diagnosis**				0.002
2013	6020 (16.0)	1428 (15.9)	4592 (16.1)	
2014	4898 (13.0)	1158 (12.9)	3740 (13.1)	
2015	5640 (15.0)	1396 (15.6)	4244 (14.8)	
2016	5487 (14.6)	1348 (15.0)	4139 (14.5)	
2017	4325 (11.5)	1048 (11.7)	3277 (11.5)	
2018	4070 (10.8)	951 (10.6)	3119 (10.9)	
2019	3879 (10.3)	821 (9.2)	3058 (10.7)	
2020	3244 (8.6)	810 (9.0)	2434 (8.5)	

*Abbreviations*: *AD* Alzheimer’s disease, *ADRD* Alzheimer’s disease and related dementia, *CCI* Charlson Comorbidity Index, *CNS* central nervous system, *SD* standard deviation

### Disease comparison cohorts

To assess whether the prevalence and secular trends in CNS-active medication polypharmacy differed between patients with vs without EOD, we selected three disease cohorts— chronic pain, depression, and epilepsy —among patients without EOD as comparisons. Those three diseases were selected as comparisons because they are managed by one of the assessed CNS-active medications (i.e., opioids for chronic pain; antidepressants for depression, and anticonvulsants for epilepsy). Similar aforementioned eligibility criteria were applied in the selection of study patients with each of the three disease conditions from the 2012–2021 MarketScan data. We included all eligible patients with epilepsy and a random 5% eligible sample with chronic pain or depression to facilitate computation. The diagnostic codes for the three conditions as comparison cohorts are given in Table A. 1.

### CNS-Active medication polypharmacy

Prescription CNS-active medications captured through MarketScan pharmacy files included antipsychotics, antidepressants, benzodiazepines, anticonvulsants, opioids, and Z-drugs, all of which are listed in the AGS Beers Criteria as drugs considered potentially inappropriate when used concurrently [14, 15] (Table A. 2). For each CNS therapeutic class, we excluded injectable drugs primarily used in inpatient settings, for which prescription dispensing claims are unavailable. In EOD and non-EOD cohorts, we reported the primary outcome of interest—CNS-active medication polypharmacy, defined as concurrent use of three or more CNS medications of interest that overlapped for greater than 30 consecutive days measured during the 12 months after the indexed disease diagnosis. The cutoff of > 30 days to define CNS polypharmacy has been used in a previous study [17]. Similar to a prior study assessing CNS-active polypharmacy for patients with LOD [17], we identified daily exposure of each CNS-active medication according to the fill date and days’ supply of the drug. For each CNS medication, we allowed a gap of 7 days or less between prescription fills to account for potential delays in refills.

**Table 2:** Combinations of CNS-active medications concurrently used among adults with early-onset dementia who had CNS Polypharmacy

Additional medication class	Percentage of 8,960 patients with CNS-active medication polypharmacy						
	**Overall**^**a**^	**Antidepressant**^**b**^	**Benzodiazepine**^**b**^	**Anticonvulsant**^**b**^	**Antipsychotic**^**b**^	**Opioid**^**b**^	**Z-drug**^**b**^
**Antidepressant**	93.7	60.9	56.1	44.2	44.4	37.0	18.5
**Benzodiazepine**	57.2		11.2	39.2	44.2	36.0	18.2
**Anticonvulsant**	46.4			24.4	39.2	35.2	15.0
**Antipsychotic**	45.0				16.9	25.7	14.6
**Opioid**	38.3					25.8	20.2
**Z-drug**	19.3						2.3

*Abbreviations*: *CNS* central nervous system

^a^Percentages of 10,970 patients with CNS-active medication polypharmacy with co-use of a specific therapeutic class of CNS medications

^b^Percentages of 10,970 patients with CNS polypharmacy prescribed a specific combination of CNS-active medications within or between therapeutic classes. A patient may have more than one drug combination

### Covariates

In EOD and non-EOD disease cohorts, we measured covariates 12 months before the indexed disease diagnosis. These covariates included demographic characteristics, select clinical conditions treated through the studied CNS-active medications, and overall comorbidity. Demographic characteristics included age (categorized as < 44, 46–50, 51–54, 55–60, and 61–64 years), sex, rural residency (yes vs no), and US geographic region (categorized as Northeast, Northcentral, South, and West). We used *ICD-9-CM* and *ICD-10-CM* codes or Clinical Classification Software for these codes developed by the Healthcare Cost and Utilization Project [27] to identify clinical conditions, including diagnosis of depression, psychiatric disorder, chronic pain, seizure disorder, substance use disorder, and fall injury, that may require treatment of the studied CNS-active medications. We also measured the diagnosis of behavioral symptoms, which are prevalent among patients with ADRD and often trigger the use of CNS-active medications [28]. To assess the overall comorbidity, we calculated the Charlson Comorbidity Index for each patient by measuring the presence of 15 conditions (excluding dementia; Table A. 1**)** [29]. In EOD cohort, we also examined subtypes of the indexed ADRD diagnosis, including frontotemporal dementia, dementia with Lewy bodies, vascular dementia, Alzheimer’s disease/senile dementia, and dementia unspecified (Table A. 1) [17].

### Statistical analysis

We assessed baseline covariates for patients with EOD overall and for patients with or without CNS-active medication polypharmacy. For patients with EOD who had CNS-active medication polypharmacy, we reported the mean duration of CNS-active medication polypharmacy and combinations of CNS-active medications within or between therapeutic classes that were concurrently dispensed in a 12-month period.

We reported annual crude prevalence of CNS polypharmacy from 2013 to 2020 among patients with EOD and the three non-EOD disease cohorts (chronic pain, depression, and epilepsy). For each cohort, we used a multivariable modified Poisson regression model to examine the time trend in CNS-active medication polypharmacy. To test secular trends, we included each calendar year of indexed disease as a dummy variable in the models. The coefficients of those yearly dummy variables represented changes in the proportion of patients with CNS-active medication polypharmacy for a given year compared with the reference year of 2013. We expressed associations as prevalence relative ratios (PRRs) and their respective 95% confidence intervals (CIs). To compare differences in time trends of CNS-active medication polypharmacy between patients with EOD and each of the non-EOD disease cohorts, we tested the interaction of disease type and calendar year in a separate multivariate modified Poisson regression model.

None of the reported baseline covariates, key exposure, and outcome variables had missing values. All analyses were performed using SAS, version 9.4 (SAS Institute Inc), and all tests were two-sided with statistical significance set at *P* < 0.05.

## Results

This multi-year, cross-sectional study identified 37,563 patients with EOD (mean [SD] age, 55.9 [7.0] years; 21,284 [56.7%] females) from 2013 to 2020 (Table 1). Less than 5% of the sample had a diagnosis of dementia with Lewy bodies or frontotemporal dementia, 8.3% had vascular dementia, 14% had AD/senile dementia, and 73.1% had dementia unspecified on the index date. Among patients with EOD, 8,960 (23.9%) had concurrent use of three or more CNS-active medications for > 30 days within 12 months after a medical encounter with ADRD.

Patients with (vs without) CNS-active medication polypharmacy were more often female (64.0% vs 54.4%, *P* < 0.001), aged 55 to 65 years (66.7% vs 65.8%, *P* = 0.019), and residing in the US South (50.2% vs 44.8%, *P* < 0.001) and had received a diagnosis of the assessed clinical conditions that may require treatment of the studied CNS-active medications, including depression (78.7% vs 38.4%, *P* < 0.001), psychiatric disorder (13.8% vs 4.9%, *P* < 0.001), behavioral symptoms (29.9% vs 16.4%, *P* < 0.001), chronic pain (59.4% vs 38.5%, *P* < 0.001), epilepsy (19.4% vs 9.0%, *P* < 0.001), injury (16.7% vs 8.5%, *P* < 0.001), and substance use disorder (21.2% vs 9.0%, *P* < 0.001). Patients with (vs without) CNS-active medication polypharmacy also differed in subtypes of ADRD diagnosis on the index date (*P* < 0.001). We also identified 130,902 patients with epilepsy, 118,526 patients with depression, and 449,596 patients with chronic pain, all of whom had no diagnosis of EOD; Table A. 3 gives their baseline demographic and clinical characteristics.

**Table 3:** Multivariable modified poisson regression analyses of time trends of central nervous system–active medication polypharmacy among adults with Early-Onset Dementia (EOD), epilepsy, depression, or chronic pain, 2013–2020

	**Prevalence relative ratio (95% CI) of CNS-active medication polypharmacy (yes vs no)**							
	**EOD (*n* = 37,563)**		**Epilepsy (*n* = 130,902)**		**Depression (*n* = 118,526)**		**Chronic pain (*n* = 449,596)**	
	**Unadjusted**	**Adjusted**	**Unadjusted**	**Adjusted**	**Unadjusted**	**Adjusted**	**Unadjusted**	**Adjusted**
**Year**								
2013	1.00 [Reference]		1.00 [Reference]		1.00 [Reference]		1.00 [Reference]	
2014	1.00 (0.93–1.07)	0.99 (0.93–1.06)	1.00 (0.97–1.04)	0.96 (0.93–0.99)	0.95 (0.91–0.99)	0.94 (0.90–0.97)	0.93 (0.90–0.97)	0.92 (0.89–0.96)
2015	1.04 (0.98–1.11)	0.95 (0.90–1.01)	1.00 (0.96–1.03)	0.90 (0.88–0.93)	0.92 (0.89–0.96)	0.87 (0.84–0.91)	0.92 (0.88–0.95)	0.85 (0.82–0.88)
2016	1.04 (0.97–1.11)	0.96 (0.90–1.01)	1.00 (0.96–1.04)	0.93 (0.90–0.96)	0.83 (0.79–0.87)	0.82 (0.79–0.86)	1.02 (0.98–1.07)	0.84 (0.81–0.88)
2017	1.02 (0.95–1.10)	0.97 (0.91–1.03)	0.92 (0.89–0.96)	0.88 (0.85–0.92)	0.77 (0.73–0.80)	0.79 (0.75–0.83)	0.96 (0.91–1.01)	0.78 (0.74–0.82)
2018	0.99 (0.92–1.06)	0.93 (0.87–1.00)	0.90 (0.86–0.94)	0.87 (0.83–0.90)	0.76 (0.72–0.80)	0.79 (0.75–0.83)	0.96 (0.91–1.01)	0.75 (0.71–0.78)
2019	0.89 (0.83–0.96)	0.83 (0.77–0.89)	0.90 (0.86–0.93)	0.85 (0.82–0.88)	0.71 (0.68–0.75)	0.74 (0.71–0.78)	0.88 (0.84–0.93)	0.66 (0.62–0.69)
2020	1.05 (0.98–1.11)	0.94 (0.88–1.01)	0.93 (0.89–0.97)	0.86 (0.83–0.89)	0.78 (0.75–0.82)	0.81 (0.77–0.85)	0.94 (0.89–0.99)	0.66 (0.63–0.69)
**Covariates**								
**Age group, y**								
≤ 44	1.00 [Reference]		1.00 [Reference]		1.00 [Reference]		1.00 [Reference]	
45–49	1.10 (1.01–1.20)	0.92 (0.85–0.99)	1.05 (1.02–1.09)	1.03 (1.00–1.06)	1.13 (1.08–1.17)	1.12 (1.08–1.17)	1.18 (1.13–1.23)	1.16 (1.11–1.20)
50–54	1.08 (1.00–1.17)	0.99 (0.93–1.06)	1.05 (1.02–1.09)	1.03 (1.00–1.06)	1.24 (1.19–1.28)	1.18 (1.14–1.23)	1.28 (1.24–1.33)	1.24 (1.19–1.28)
55–59	1.13 (1.05–1.21)	1.01 (0.95–1.08)	1.04 (1.01–1.07)	1.03 (1.00–1.06)	1.30 (1.25–1.34)	1.20 (1.16–1.25)	1.33 (1.28–1.38)	1.26 (1.22–1.31)
60–64	1.07 (1.00–1.15)	0.99 (0.93–1.05)	0.98 (0.95–1.02)	0.99 (0.96–1.02)	1.39 (1.34–1.44)	1.25 (1.21–1.30)	1.34 (1.29–1.39)	1.25 (1.20–1.29)
**Sex**								
Male	1.00 [Reference]		1.00 [Reference]		1.00 [Reference]		1.00 [Reference]	
Female	1.36 (1.31–1.41)	1.20 (1.16–1.25)	1.64 (1.60–1.68)	1.31 (1.28–1.34)	1.22 (1.19–1.25)	1.22 (1.19–1.25)	2.04 (1.99–2.10)	1.52 (1.48–1.56)
**Locale**								
Metropolitan	1.00 [Reference]		1.00 [Reference]		1.00 [Reference]		1.00 [Reference]	
Rural	1.04 (1.00–1.08)	1.01 (0.98–1.05)	1.05 (1.02–1.07)	1.00 (0.98–1.02)	0.98 (0.95–1.00)	0.97 (0.95–1.00)	1.08 (1.05–1.11)	1.02 (0.99–1.05)
**US Region**								
Northeast	1.00 [Reference]		1.00 [Reference]		1.00 [Reference]		1.00 [Reference]	
Midwest	1.27 (1.19–1.34)	1.19 (1.13–1.26)	1.24 (1.20–1.29)	1.23 (1.19–1.27)	1.15 (1.10–1.20)	1.15 (1.10–1.20)	1.35 (1.30–1.41)	1.33 (1.28–1.39)
South	1.34 (1.27–1.41)	1.30 (1.23–1.36)	1.38 (1.34–1.42)	1.32 (1.28–1.36)	1.34 (1.29–1.39)	1.32 (1.28–1.37)	1.49 (1.44–1.55)	1.48 (1.43–1.54)
West	1.15 (1.07–1.24)	1.19 (1.11–1.27)	1.20 (1.16–1.25)	1.21 (1.16–1.25)	1.17 (1.12–1.22)	1.17 (1.12–1.22)	1.22 (1.16–1.27)	1.24 (1.19–1.29)
**Clinical condition** (yes vs no)								
Depression	4.00 (3.82–4.19)	3.33 (3.18–3.49)	3.88 (3.80–3.96)	3.15 (3.08–3.22)	––	––	8.41 (8.20–8.62)	6.84 (6.66–7.02)
Psychiatric disorder	2.11 (2.02–2.21)	1.28 (1.21–1.34)	2.39 (2.30–2.48)	1.26 (1.21–1.31)	2.70 (2.54–2.87)	1.60 (1.47–1.74)	7.34 (6.83–7.88)	1.68 (1.52–1.86)
Behavioral Symptoms	1.75 (1.69–1.82)	1.04 (0.99–1.08)	1.87 (1.82–1.92)	1.06 (1.03–1.09)	2.47 (2.35–2.59)	1.14 (1.07–1.22)	5.70 (5.40–6.01)	1.09 (1.01–1.17)
Chronic pain	1.90 (1.83–1.97)	1.47 (1.41–1.52)	2.05 (2.01–2.10)	1.40 (1.37–1.43)	2.06 (2.01–2.11)	1.68 (1.64–1.73)	––	––
Epilepsy	1.85 (1.78–1.93)	1.45 (1.39–1.52)	––	––	2.59 (2.47–2.73)	1.75 (1.66–1.86)	4.63(4.38–4.90)	2.01 (1.88–2.14)
Fall injury	1.73 (1.65–1.81)	1.17 (1.12–1.22)	1.79 (1.74–1.84)	1.22 (1.19–1.25)	1.61 (1.54–1.68)	1.19 (1.14–1.24)	2.09 (2.00–2.17)	1.30 (1.25–1.35)
Substance use disorder	1.99 (1.92–2.08)	1.36(1.31–1.42)	2.00 (1.95–2.05)	1.27 (1.24–1.30)	2.01 (1.95–2.08)	1.82 (1.76–1.88)	3.88 (3.75–4.02)	1.98 (1.91–2.05)
**CCI**	1.12 (1.11–1.13)	1.02 (1.01–1.02)	1.18 (1.17–1.18)	1.06 (1.05–1.06)	1.24 (1.23–1.25)	1.10 (1.09–1.11)	1.39 (1.38–1.40)	1.21 (1.20–1.22)
**Interaction model**^**a**^								
Year × Disease status	1.00 [Reference]		0.99 (0.98–0.99)	0.98 (0.98–0.99)	0.99 (0.98–0.99)	0.99 (0.98–0.99)	1.00 (1.00–1.00)	1.00 (0.99–1.00)

*Abbreviations*: *CI* confidence interval, *CCI* Charlson Comorbidity Index, *CNS* central nervous system, *EOD* early-onset dementia

^a^A separate Poisson modified regression model was constructed including the year of indexed disease diagnosis, disease status (with vs without EOD), and interaction of the year variable with disease status

Among 8,960 patients with EOD who had CNS-active medication polypharmacy, the mean (SD) duration of receiving ≥ 3 CNS-active medication classes in a 12-month follow-up was 6.9 (4.0) months. Use of antidepressants accounted for 93.7% of patients with CNS-active medication polypharmacy, followed by benzodiazepines (57.2%), anticonvulsants (46.4%), and antipsychotics (45.0%) (Table 2). Among patients with CNS-active polypharmacy, antidepressants were the most commonly prescribed class of CNS-active medications studied. Co-use of an antidepressant with another antidepressant was 60.9%, with a benzodiazepine was 56.1%, with an anticonvulsant was 44.2%, with an antipsychotic was 44.4%, and with an opioid was 37.0%. The percentages of patients with co-use of benzodiazepines with other CNS-active medication classes (except for antidepressants) were high, ranging from 11.2% to 44.2%. We also observed high percentages of patients with co-use of anticonvulsants and opioids (35.2%) and co-used of two or more medications within anticonvulsant (24.4%) and opioid (25.8%) classes for longer than 30 days.

### Prevalence and trend of CNS polypharmacy among patients with vs without EOD

**Fig. 1:**
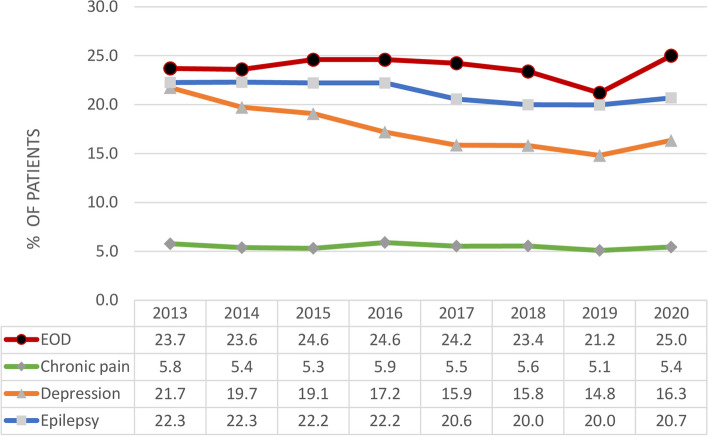
Annual Crude Prevalence of Central Nervous System–Active Medication Polypharmacy by Disease Cohort. EOD indicates, early-onset dementia

Among patients with EOD, the crude annual prevalence of CNS-active medication polypharmacy ranged from 23.7% in 2013 to 25.0% in 2021, significantly higher than the prevalence among non-EOD patients with chronic pain (5.8%–5.4%, *P* < 0.001), depression (21.7%–16.3%, *P* < 0.001), or epilepsy (22.3%–20.7%, *P* < 0.001) (Fig. 1). After adjustment for covariates, the annual prevalence of CNS medication polypharmacy did not significantly change between 2013 and 2020 (adjusted PRR [aPRR], 0.94; 95% CI, 0.88–1.01). By contrast, a significant decreasing trend in the prevalence of CNS medication polypharmacy was observed among patients without EOD who had a diagnosis of epilepsy (aPRR, 0.86; 95% CI, 0.83–0.89), depression (aPRR, 0.81; 95% CI, 0.77–0.85), or chronic pain (aPRR, 0.66; 95% CI, 0.63–0.69) between 2013 and 2021 (Table 3). The interaction analysis indicated that patients with epilepsy (aPRR, 0.98 [95% CI, 0.98–0.99]; *P* = 0.012 for interaction) or depression (aPRR, 0.99 [95% CI, 0.98–0.99]; *P* < 0.001 for interaction) vs with EOD had decreasing probability of CNS polypharmacy over time (Table 3).

## Discussion

This multi-year, cross-sectional study using a US national commercial insurance claims dataset is among the first to provide population-based data on the prevalence and trend of CNS-active medication polypharmacy among patients with EOD across a recent 9-year (2012–2021) period. Our first key finding was a high prevalence of CNS-active medication polypharmacy among patients with EOD, with one in four patients simultaneously using three or more CNS-active medications on the AGS Beers Criteria list for longer than 30 days. Our second key finding was that the annual prevalence of CNS-active medication polypharmacy during the study period was higher in general among patients with EOD vs patients without EOD but having epilepsy, depression, or chronic pain, conditions often managed by one of the studied CNS-active therapeutic drug classes. The third key finding was that the secular trend of CNS-active polypharmacy remained unchanged between 2013 and 2020 among patients with EOD, whereas a significant decreasing trend was observed in each of the three non-EOD disease groups. Our findings suggest that CNS-active medication polypharmacy was more common and remained high over time among adults with EOD vs without, raising safety concerns among this understudied adult population who developed dementia during their prime working-age (between 30 and 64) years.

The high prevalence of CNS-active medication polypharmacy (23.7% in 2013 to 25.0% in 2021) observed among patients with EOD was nearly 1.8-fold as high as the prevalence (13.9% in 2018) of CNS-active medication polypharmacy observed in a prior study of patients with LOD [17]. The higher prevalence of CNS-active medication polypharmacy among patients with EOD (vs with LOD) is likely because the former group had a higher prevalence of seizures [6] and neuropsychiatric symptoms [7], leading to increased use of anticonvulsants and antidepressants, two commonly prescribed therapeutic classes of CNS-active medications observed in the study sample with EOD. Also, patients with EOD tended to have substance abuse and traumatic brain injury [8], risk factors associated with the use of multiple CNS-active medications observed in the present and previous studies [30]. The present study also observed a high percentage (43.5%) of patients with EOD who had a comorbid chronic pain diagnosis, a condition that strongly predicts the use of multiple CNS-active medications [18]. Overall, our findings indicate that patients with EOD are at higher risk than patients with LOD for receiving CNS-active medication polypharmacy.

Our study found that antidepressants are the most common CNS medication that contributes to CNS polypharmacy among patients with EOD, consistent with findings among patients with LOD [17]. Our study, however, found that benzodiazepines were the second most prescribed CNS-active medication, occurring in over half (57.2%) of patients with EOD; an estimate substantially higher than that observed among patients with LOD (40.7%) [17]. Furthermore, co-use of opioids and anticonvulsants and co-use of the same medications within these two CNS medication classes were also high among patients with EOD. The prevalent co-use of prescription opioids and anticonvulsants may reflect changes in the prescribing practice of pain medications in recent years [31] given that clinical pain guidelines recommend combined use of opioids with non-opioid analgesics, such as anticonvulsants, for pain management to reduce opioid use and related harms [32]. Nevertheless, the combined use of these CNS-active medications has been associated with serious adverse events, including opioid overdose [33], opioid-related death [34], and falls [35]. Closely monitoring patients with co-use of benzodiazepines, anticonvulsants, and opioids is warranted.

Another important finding of the present study is that the secular trend of CNS-active medication polypharmacy was unchanged among patients with EOD, whereas a significant decreasing trend was observed for the three disease cohorts (i.e., chronic pain, depression, and epilepsy) without EOD. Our finding of decreasing trends in CNS polypharmacy among the three non-EOD adult patients appears encouraging, suggesting that both clinicians and patients may have increased their attention to reducing CNS-active polypharmacy during the past decade. Nevertheless, no decreasing trend was observed among adult patients with EOD. The reasons for this observation are unclear and could be multifaceted. Patients with EOD have been largely understudied. Our finding calls for further research investigations toward understanding clinical reasons for patients with EOD receiving multiple CNS-active medications and associated benefits (e.g., improved neuropsychiatric symptoms) and risks (e.g., injury) in the management of EOD and co-occurring conditions that require CNS medications.

The present study provides referential data for clinicians to understand that CNS-active medication polypharmacy was common in a representative sample of US commercially insured adults and remained high among patients with EOD across the past decade. Clinical attention is needed in reviewing the medications of adults with EOD who have CNS-active medication polypharmacy, particularly those with the strong risk factors observed in the present study, to ensure that the benefits outweigh the risks for combined use of these treatments.

## Strengths and Limitations

Several strengths of this study are noteworthy. Our study complements the existing literature by adding to the understanding of the prevalence and trend of CNS-active medication polypharmacy among patients with EOD in the most recent decade. The use of large administrative claims data yielded a sufficient sample of adults with EOD. Using nearly a decade of accumulated data representing the US commercially insured population allowed for the understanding of secular trends in CNS-active medication polypharmacy. Finally, the use of three non-EOD disease groups enabled the comparison of CNS-active medication polypharmacy, clarifying whether this potentially inappropriate prescribing practice differed between patients with vs without EOD.

Several study limitations warrant acknowledgment. First, we relied on reimbursed pharmacy dispensing records, which provided information on prescription drugs dispensed but not consumed and lacked information on whether medications were given as needed or around the clock. Such information could lead to inaccurate estimates of CNS-active medication polypharmacy defined as concomitant use of three or more CNS-active medications of interest. To address this limitation, we required dispensed prescription concomitancy for > 30 consecutive days to ensure concomitant use of these CNS-active medications. Second, our claims data had no information on race and ethnicity, income and educational levels, and other important risk factors (e.g., health status in general) for EOD that may have further informed our multivariable models. Third, the pharmacy dispensing records did not capture prescriptions paid out of pocket, which may lead to an underestimation of CNS-active medication polypharmacy. Fourth, claims-based data have limited accuracy in identifying individuals with ADRD given that the disease is often under or delayed diagnosed [36]. Finally, our study results are generalizable only to privately insured populations with continuous coverage for at least 12 months before and after an ADRD diagnosis and may not extend to patients covered by public insurance or those who are uninsured. Our data also cannot confirm whether the ADRD diagnosis occurred in the employed person or their caregiver.

## Conclusion

Among US commercially insured adults with EOD, one in four had concurrent use of three or more CNS-active medications for longer than 30 days in a given year between 2013 and 2020. The prevalence of CNS-active medication polypharmacy among patients with EOD (vs without EOD) was higher and remained unchanged across the 9-year period. Our findings call for research priorities toward understanding reasons for patients with EOD receiving CNS polypharmacy and the associated benefits and risks. Clinical attention in reviewing medications for patients with EOD who have CNS-active medication polypharmacy is also needed.

### Supplementary Information

**Additional file1 :** **Table A.1.** ICD-9-CM and ICD-10-CM Codes for Disease Conditions Considered in the Study. **Table A.2.** Central Nervous System (CNS) Medications Considered in the Study. **Table A.3.** Demographic and Clinical Characteristics of Commercially Insured Adults with Epilepsy, Depression, or Chronic Pain but with no ADRD.

## Data Availability

No datasets were generated or analysed during the current study.
