# Supplementary material for: Prevalence and trend of central nervous system–active medication polypharmacy among US commercially insured adults with vs without early-onset dementia: a multi-year cross-sectional study
Source: Alzheimers Res Ther. 2024 Feb 8;16:30. doi: 10.1186/s13195-024-01405-y (PMC10851564; doi:10.1186/s13195-024-01405-y)
Supplement: Supplementary file 1 — Additional file1 : Table A.1. ICD-9-CM and ICD-10-CM Codes for Disease Conditions Considered in the Study. Table A.2. Central Nervous System (CNS) Medications Considered in the Study. Table A.3. Demographic and Clinical Characteristics of Commercially Insured Adults with Epilepsy, Depression, or Chronic Pain but with no ADRD. [file 13195_2024_1405_MOESM1_ESM.docx]

**Table A.1:** *ICD-9-CM and ICD-10-CM* Codes for Disease Conditions Considered in the Study

| **Disease, Condition, or Medication** | ***ICD-9-CM, ICD-10-CM* code, or Medication** | **Algorithm** |
| --- | --- | --- |
| ADRD | 290.0, 290.1, 290.10, 290.11, 290.12, 290.13, 290.2, 290.20, 290.21, 290.3, 290.4, 290.40, 290.41, 290.42, 290.43, 294.0, 294.1, 294.10, 294.11, 294.2, 294.20, 294.21, 294.8, 331.0, 331.1, 331.11, 331.19, 331.2, 331.7, 331.82, 797, F03.90, F01.50, F01.51, F04, F02.80, F02.81, F03.90, F03.91, F06.1, F06.8, G30.0, G30.1, G30.8, G30.9, G31.01, G31.09, G31.0, G31.1, G94, G31.83, R41.81  Subtypes:  Dementia with Lewy bodies: 331.82, G31.83  Frontotemporal dementia: 331.1, 331.11, 331.19, G31.01, G31.09, G31.0  Vascular dementia: 290.4, 290.40, 290.41, 290.42, 290.43, F01.50, F01.51  Alzheimer’s disease/senile dementia: 331.0, G30.0, G30.1, G30.8, G30.9  Dementia unspecified: any other ICD-9 or ICD-10 codes above that are not listed | At least 1 inpatient or outpatient claim with a disease code in any diagnostic position |
| Chronic pain |  |  |
| Musculoskeletal | 274.x, 710.x-729.x (exclude 723.4, 724.3, 724.4, 729.1, 729.2), A18.01-A18.02, A52.16, D48.1, E08.61x, E09.61x, E10.61x, E11.61x, E13.61x, M00-M02, M04.02-M04.09, M05-M19, M1A, M20.10, M21.61-M21.62, M22-M25, M32-M36, M43.2-M43.8x9, M45-M48, M49.8x, M50.xx-M51.xx, M53.xx, M54.xx, M60.0-M60.2, M61-M63, M65-M67, M70-M72, M75-M77, M79, M96.1, M99.xx, N20.0, Q68.6, R25.2, R26.2, R29.8x | At least 1 inpatient or outpatient claim with a disease code in any diagnostic position |
| Neuropathic | 053.1x, 249.6, 250.6, 307.89, 336.x, 337.x, 338.0, 338.4, 340, 350.x, 351.x, 352.1, 353.x-355.x, 357.1, 357.2-357.4, 357.8, 357.9, 723.4, 724.3, 724.4, 729.1, 729.2, A52.15, B02 (exclude B02.1), EXX.4, EXX.610, EXX.65 (where X in “08”-“13”), E10.4, F45.42, G13.0, G13.1, G32.0, G35, G50- G52.1, G54-G59, G61.8, G61.9, G62.8, G62.9, G63-G65, G89.0, G90.52, G90.59, G90.51, G95.9, G95.89, G99.2, M05.51, M05.52, M05.53, M05.54, M05.55, M05.56, M05.57, M54.13-M54.18, M54.1-M54.5, M60.8, M60.9, M79.1, M79.2, M79.7 |  |
| Idiopathic | 338.2x, 338.4, 780.96, G89.4, R52 |  |
| Depression | 296.2x, 296.3x, 296.5x (where x in “1”-“6”), 296.6x, 296.89, 298.0, 298.0, 300.4, 309.1, 311, F31.3x, F31.5, F31.6x, F31.7x (where x in “5”-“8”), F31.81, F32.x, F33.x, F33.41, F33.42, F33.8, F33.9, F34.1, F43.21, F43.23 | At least 1 inpatient or outpatient claim with a disease code in any diagnostic position |
| Epilepsy | 345, 345.x, 345.xx, G40.00x, G40.01x, G40.10x, G40.11x, G40.20x, G40.21x, G40.30x, G40.31x, G40.40x, G40.41x, G40.42, G40.50x, G40.80x, G40.81x, G40.82x, G40.83x, G40.89, G40.90x, G40.91x, G40.A0x, G40.A1x, G40.B0x, G40.B1x |  |
| Substance use disorder | Substance-related disorders (CCS 661), Alcohol-related disorders (CCS 660) |  |
| Behavioral symptom | 290.xx (exclude 290.0, 290.1, 290.10, 290.4x), 293.xx, 294.9, 294.0, 294.21, 294.11, 297.x, 298.x, 312.9, 780.97, F02.81, F03.9x, F04, F05, F06.x, F22-24, F28, F29, F32.3, F33.3, F44.89, F53, R41.82, F91.9 |  |
| Fall injury | Fall (CCS 2603), Pathological fracture (CCS 207), Fracture of neck of femur (hip) (CCS 226), Skull and face fractures (CCS 228), Fracture of upper limb (CCS 229), Fracture of lower limb (CCS 230), Other fractures (CCS 231) |  |
| Psychiatric disorder | Schizophrenia, and other psychotic disorders (CCS 659) |  |
| CCI conditions |  |  |
| Cancer | Cancer of head and neck (CCS11), Cancer of esophagus (CCS12), Cancer of stomach (CCS13), Cancer of colon (CCS14), Cancer of rectum and anus (CCS15), Cancer of liver and intrahepatic bile duct (CCS16), Cancer of pancreas (CCS17), Cancer of other GI organs, peritoneum (CCS18), Cancer of bronchus lung (CCS19), Cancer, other respiratory and intrathoracic (CCS20), Cancer of bone and connective tissue (CCS21), Melanomas of skin (CCS22), Other non-epithelial cancer of skin (CCS23), Cancer of breast (CCS24), Cancer of uterus (CCS25), Cancer of cervix (CCS26), Cancer of ovary (CCS27), Cancer of other female genital organs (CCS28), Cancer of prostate (CCS29), Cancer of testis (CCS30), Cancer of other male genital organs (CCS31), Cancer of bladder (CCS32), Cancer of kidney and renal pelvis (CCS33), Cancer of other urinary organs (CCS34), Cancer of brain and nervous system (CCS35), Cancer of thyroid (CCS36), Hodgkin`s disease (CCS37), Non-Hodgkin`s lymphoma (CCS38), Leukemias (CCS39), Multiple myeloma (CCS40), Cancer, other and unspecified primary (CCS41), Secondary malignancies (CCS42), Malignant neoplasm without specification of site (CCS43) | HCUP CCS for *ICD-9-CM or ICD-10-CM* |
| Myocardial Infarction | CCS 100 |  |
| Congestive Heart Failure | CCS 108 |  |
| Peripheral vascular | Peripheral and visceral atherosclerosis (CCS 114), Aortic; peripheral; and visceral artery aneurysms (CCS 115), Aortic and peripheral arterial embolism or thrombosis (CCS116) |  |
| Cerebrovascular Disease | acute cerebrovascular disease (CCS 109), Other and ill-defined cerebrovascular disease (CCS 111), Late effects of cerebrovascular disease (CCS113) |  |
| Pulmonary Disease | Chronic obstructive pulmonary disease and bronchiectasis (CCS 127) |  |
| Peptic Ulcer Disease | CCS139 |  |
| Diabetes mellitus without complication | CCS 49 |  |
| Diabetes mellitus with complications | CCS 50 |  |
| Renal Disease | Nephritis; nephritis; renal sclerosis (CCS 156), acute and unspecified renal failure (CCS 157), chronic kidney disease (CCS 158), other diseases of kidney and ureters (CCS 161) |  |
| Liver Disease | CCS 151 |  |
| Paraplegia and Hemiplegia | CCS 82 |  |
| HIV | CCS 5 |  |
| Connective Tissue Disease | CCS 211 |  |
| Rheumatoid arthritis and related disease | CCS 202 |  |

Abbreviations: ADRD, Alzheimer disease and related dementia; CCI, Charlson Comorbidity Index; CCS, Clinical Classification Software; CNS, central nervous system; HCUP, Healthcare Cost and Utilization Project; *ICD-9-CM and ICD-10-CM*, *International Classification of Diseases, Ninth or Tenth Revision, Clinical Modification*.

**Table A.2:** Central Nervous System (CNS) Medications Considered in the Study

| **Medication class** | **CNS-active medications** |
| --- | --- |
| Antipsychotic | acetophenazine, chlorpromazine, droperidol, fluphenazine, haloperidol, loxapine, molindone, perphenazine, pimozide, prochlorperazine, thioridazine, thiothixene, trifluoperazine, aripiprazole, asenapine, brexpiprazole, cariprazine, clozapine, iloperidone, fanapt, lurasidone, olanzapine, paliperidone, primavaserin, quetiapine, risperidone, ziprasidone |
| Antidepressant | mirtazapine, bupropion, isocarboxazid, phenelzine, tranylcypromine, selegiline, rasagiline, nefazodone, trazodone, duloxetine, venlafaxine, desvenlafaxine, milnacipran, levomilnacipran, citalopram, escitalopram, fluoxetine, fluvoxamine, paroxetine, sertraline, vilazodone, vortioxetine, amitriptyline, clomipramine, imipramine, doxepin, trimipramine, amoxapine, maprotiline, nortriptyline, desipramine, protriptyline |
| Anticonvulsant | acetazolamide, brivaracetam, carbamazepine, cenobamate, eslicarbazepine acetate, ethadione, ethotoin, ethosuximide, ezogabine, felbamate, fenfluramine, fosphenytoin, gabapentin, lacosamide, lamotrigine, levetiracetam, mephenytoin, methohexital, methsuximide, magnesium sulfate, oxcarbazepine, perampanel, phenytoin, paramethadione, pregabalin, phenobarbital, primidone, rufinamide, stiripentol, sultiame, tiagabine, topiramate, trimethadione, valproate/divalproex, valproic acid, vigabatrin, zonisamide |
| Opioid | butorphanol, buprenorphine, codeine, dihydrocodeine, fentanyl, hydrocodone, hydromorphone, levorphanol, meperidine, methadone, morphine, nalbuphine, opium, oxycodone, oxymorphone, pentazocine, remifentanil, sufentanil, tapentadol, tramadol |
| Benzodiazepine | alprazolam, estazolam, lorazepam, oxazepam, temazepam, triazolam, midazolam, chlordiazepoxide, clobazam, clonazepam, clorazepate, diazepam, prazepam, flurazepam, quazepam |
| Z-drug | eszopiclone, zaleplon, zolpidem |

**Table A.3** Demographic and Clinical Characteristics of Commercially Insured Adults with Epilepsy, Depression, or Chronic Pain but with no ADRD.

| **Characteristic** | **Epilepsy**  **No. (%)** | **Depression^*^**  **No. (%)** | **Chronic Pain^*^**  **No. (%)** |
| --- | --- | --- | --- |
| **Overall** | 130,902 (100) | 118,528 (100) | 449,596 (100) |
| **Age, y** |  |  |  |
| Mean (SD) | 51.2 (8.3) | 50.6 (8.1) | 51.3 (8.2) |
| ≤44 | 32,372 (24.7) | 31,784 (26.8) | 109,756 (24.1) |
| 45-49 | 21,245 (16.2) | 20,900 (17.6) | 73,618 (16.4) |
| 50-54 | 24,319 (18.5) | 21,983 (18.5) | 84,877 (18.9) |
| 55-59 | 26,259 (20.6) | 22,993 (19.4) | 90,612 (20.2) |
| 60-64 | 26,707 (20.4) | 20,866 (17.6) | 90,733 (20.2) |
| **Sex** |  |  |  |
| Male | 55,167 (42.1) | 36,112 (30.5) | 196,366 (43.7) |
| Female | 75,735 (57.9) | 82,414 (69.5) | 253,230 (56.3) |
| **Locale** |  |  |  |
| Metropolitan | 94,963 (72.6) | 83,934 (70.8) | 328,866 (73.1) |
| Rural | 35,939 (27.5) | 34,592 (29.2) | 120,730 (26.9) |
| **US Region** |  |  |  |
| Northeast | 24,965 (19.1) | 19,834 (16.7) | 78,058 (17.4) |
| Midwest | 27,107 (20.7) | 26,394 (22.3) | 93,309 (20.8) |
| South | 58,910 (45.0) | 51,832 (43.7) | 199,012 (44.3) |
| West | 19,920 (15.2) | 20,466 (17.3) | 79,217 (17.6) |
| **Clinical condition** (Yes vs No) |  |  |  |
| Depression | 38,528 (29.4) | -- | 79,900 (17.8) |
| Psychiatric disorder | 2927 (2.2) | 1168 (1.0) | 1178 (0.3) |
| Behavioral symptoms | 12,107 (9.3) | 2217 (1.9) | 3202 (0.7) |
| Chronic pain | 48,235 (36.9) | 38,502 (32.5) | -- |
| Epilepsy | -- | 1855 (1.6) | 4012 (0.9) |
| Injury | 10,828 (8.3) | 6207 (5.2) | 20,650 (4.6) |
| Substance use disorder | 12,518 (9.6) | 9620 (8.1) | 16,063 (3.6) |
| CCI (Mean± SD) | 1.3±1.5 | 1.0±1.1 | 0.9±1.1 |
| **Year of Indexed Disease Diagnosis** |  |  |  |
| 2013 | 27,960 (21.4) | 23,716 (20.0) | 121,995 (27.1) |
| 2014 | 18,590 (14.2) | 16,239 (13.7) | 81,497 (18.1) |
| 2015 | 17,565 (13.4) | 16,423 (13.9) | 74,642 (16.6) |
| 2016 | 15,297 (11.7) | 13,440 (11.3) | 37,728 (8.4) |
| 2017 | 12,319 (9.4) | 11,082 (9.3) | 32,096 (7.1) |
| 2018 | 12,966 (9.9) | 11,668 (9.8) | 34,261 (7.6) |
| 2019 | 13,813 (10.6) | 13,064 (11.0) | 35,165 (7.8) |
| 2020 | 12,392 (9.5) | 12,894 (10.9) | 32,212 (7.2) |

Abbreviations: ADRD, Alzheimer’s disease and related dementia; CNS, central nervous system; CCI, Charlson Comorbidity Index.

^*^We included all eligible samples with epilepsy and a random 5 percent eligible sample with chronic pain or depression to facilitate computation.
